# The Role of Resilience in the Sibling Experience of Pediatric Palliative Care: What Is the Theory and Evidence?

**DOI:** 10.3390/children5070097

**Published:** 2018-07-16

**Authors:** Wei Ling Chin, Tiina Jaaniste, Susan Trethewie

**Affiliations:** 1 Department of Palliative Care, Sydney Children’s Hospital, Randwick, NSW 2031, Australia; weiling.chin@student.unsw.edu.au (W.L.C.); Susan.Trethewie@health.nsw.gov.au (S.T.); 2 School of Women’s and Children’s Health, University of New South Wales, Kensington, NSW 2033, Australia

**Keywords:** resilience, sibling, life-limiting condition, palliative care, functioning

## Abstract

Siblings of children with life limiting conditions (LLC) are an important part of the broader family system and require consideration in the holistic care of the family. There can be considerable variation in the functioning and adjustment of these siblings. The current paper explores the resilience paradigm, particularly in the context of siblings of children with LLC and serious medical conditions. The potential impact of children living with a seriously ill brother or sister will be overviewed, and a range of functional outcomes considered. Factors contributing to sibling resilience are detailed, including individual, family, and broader external and social factors. Given the limited research with siblings of children with LLC, literature has also been drawn from the siblings of children with serious and/or chronic medical conditions. Implications for clinical practice and future research are considered. Pediatric palliative care services may be well placed to contribute to this body of research as they have commonly extended relationships with the families of children with LLC, which span across the child’s disease trajectory.

## 1. Introduction

Life-limiting conditions (LLC) are medical conditions for which there is no reasonable hope for cure, ultimately resulting in premature death [1]. In pediatric palliative care, LLCs are considered to be those in which health professionals expect the child may die before reaching adulthood. Children with LLC have a wide range of diagnoses, including cerebral palsy, malignancy, epilepsy, and neuromuscular conditions [2], as well as a variety of rare congenital conditions. The experience of children with LLC cannot be considered in isolation, but rather within a broader family system. Thus, it is increasingly recognized that families should be considered as the unit of care [3,4]. About 80% of a sample of researchers and clinicians working in the field of pediatric palliative care who were surveyed in a Delphi Poll identified the needs of siblings as being an important area for future research [5]. However, to date, the experience of siblings growing up with a child with an LLC has received relatively little research attention and yet these well siblings may face significant challenges pervading all aspects of their family life, often also impacting on the psychological, social, and academic functioning.

Among siblings of children with a serious medical condition or LLC, there is evidence of considerable variation in functioning and adjustment [6,7]. However, it is not well understood why some siblings respond in a more adaptive, resilient way. The current paper will begin with a theoretical overview of the resilience paradigm, giving particular consideration to the context of having a sibling with a serious medical condition. Prior to a comprehensive exploration of individual, familial, and social factors that may promote sibling resilience, the paper will outline the nature of the challenges faced by siblings of children with a serious medical condition, as well as how sibling functioning is commonly assessed. Given the limited research that has been carried out in the content of siblings of children with LLC, key findings will also be drawn from the context of siblings of children with other serious illnesses and chronic conditions. The paper will conclude with some suggested directions for future research and tentative applications for clinical practice.

## 2. The Construct of Resilience

Resilience is commonly defined as the ability to maintain functionality or normal development, despite significant adversities that have the potential to disturb the normal processes in life. Thus, the construct of resilience entails two key elements: (1) the presence of adverse life events or ongoing situations, and (2) the maintenance of normal functioning [8,9]. In order to understand an individual’s resilience, it is important to have sound knowledge of both elements.

Resilience is a dynamic process that involves complex interactions between existing resources, such as personality traits, family factors, and external supports, which aids in an individual’s resistance against adverse situation or experience [10,11]. It is generally recognized in the current theoretical literature that the resilience of a child or adolescent pertains to his or her ability to navigate towards health-sustaining resources, such as the acquisition of coping strategies or external supports [12]. It thus requires the presence or availability of health-sustaining resources, which may depend on the family, social, and cultural context in which the child is functioning [12]. Hence, resilience should not be considered inherent to an individual, but rather dependent on the individual, as well as the broader context in which the individual is functioning.

## 3. Potential Impacts on Siblings’ Daily Lives

When considering an individual’s resilience, it is important to consider the nature of the adverse situation with which they are confronted. Not all adversities interact with protective factors in the same way [13]. Having a child with a LLC or a serious medical condition is likely to have a huge impact on the family unit. Depending on the level of disability of the sick child, healthy siblings may have their daily lives compromised in various ways [14]. Routines within the family commonly emerge to meet the physical needs of the unwell child, often revolving around complex treatment regimens and medical appointments. Parental time, energy, and attention is often devoted to the needs of the sick child, leaving fewer physical and emotional resources for supporting other well siblings with their everyday tasks and needs, such as homework, hobbies, and social activities [6,15,16]. Moreover, older well siblings may also take on greater caring responsibilities, with possible expectations of greater self-sufficiency [16]. Parents who are highly stressed about the sick child may be less able to recognize and respond to the needs of their well children, resulting in poorer parent–sibling communication and interactions [7,15,17]. This may result in siblings feeling misunderstood, isolated, and excluded. Furthermore, the impact of a child’s medical condition on well siblings may be greater if there are more visible signs of illness or disability in the sick child. Visible disability is likely to be associated with greater stigma and thus have a greater impact on the social interactions of the healthy child [18].

## 4. The Functional and Developmental Outcomes of Siblings

When considering the resilience of an individual in the face of an adverse situation or experience, it is by definition necessary to consider their functioning and/or development. Where possible, it is important to consider more than just one or two functional or developmental outcomes, given that an individual’s ability to function across different domains may vary markedly [10]. An individual may show resilience in some aspects of functioning, but not in others.

In the context of having a seriously ill sibling, some of the most commonly assessed domains of functioning include internalizing behaviors (such as depressive mood or elevated anxiety), externalizing behaviors (such as behavioral problems), social competence, school functioning, and academic performance [16,17,18,19,20,21,22,23,24]. In research contexts, these domains of functioning are typically assessed using validated questionnaires. Clinically, psychometric measures may also be useful, but should be used in conjunction with a thorough interview with the siblings and parents, followed up with regular monitoring. A greater proportion of siblings of unwell children have been found to fall into at-risk or clinical ranges on measures of behavioral problems, anxiety, or depression, relative to normative samples [25]. A number of studies have found externalizing problems, such as aggression and delinquency, to be more common amongst siblings of children with an LLC or serious medical condition [17,26].

Although many studies have found healthy siblings of seriously ill children to have difficulties in some aspects of functioning [17,19,21,22,26], other studies have not found any such ill effects [15,18,27,28]. Indeed there is a body of theoretical and empirical literature that has explored the concept of post traumatic growth [29,30], whereby some individual who faces adverse and/or traumatic experiences may grow or develop in some positive way. For example, it has been reported that some siblings who have a brother or sister with a serious medical condition may develop positively in certain respects, such as increased maturity and a sense of responsibility, greater patience, greater empathy, and closer family relationships [15,25,28]. In light of the heterogeneity of outcomes [6], whereby some siblings have poorer functioning on certain measures, whereas others do not, there is a need for careful examination of factors that may promote resilience or even growth.

## 5. Resilience Factors

Factors contributing to an individual’s resilience may, broadly, be grouped into three categories: (1) individual factors (e.g., trait optimism, positive affect, and psychological flexibility); (2) family functioning factors (e.g., parental adjustment, family communication, sibling relationship, and socioeconomic status); and (3) external support (e.g., social support, use of respite services). Where possible, the theoretical and empirical literature will be reviewed for each of these types of resilience factors, giving particular consideration to the context of children and adolescents with a brother or sister with a serious medical condition or LLC.

### 5.1. Individual Resilience Factors

Individual factors with the potential to promote resilience may be stable, trait-like resources (e.g., trait optimism, trait positive affect, trait mindfulness), or more dynamic and modifiable mechanisms (e.g., acceptance, psychological flexibility) [31]. Individual resilience resources and mechanisms have been comprehensively reviewed in the context of pediatric chronic pain [31]. However, we know from the theoretical literature that resilience is context-specific [12,13]. Therefore, a review of these factors is needed in the context of siblings of children with serious medical conditions or LLCs.

Trait optimism is a positive mental, future-oriented attitude, reflecting hope of a favorable outcome even amidst adversities. Optimism is likely to be helpful across a broad range of contexts by serving as a buffer against potentially negative experiences. Moreover, it is thought that optimism may have indirect positive health effects by promoting protective coping strategies and health behaviors [32]. Optimism is widely regarded as a general individual difference variable, not dependent on circumstances or settings [33]. Nevertheless, there is some literature to suggest that optimism may be taught (e.g., [34]).

Siblings of pediatric oncology patients who have reported the development of understanding, empathy, and optimism throughout the disease progression have reported finding that this helped them cope during difficult situations [28]. Sibling optimism in the context of having a brother or sister with childhood cancer has been found to be associated with lower levels of anxiety, insecurity, and loneliness in siblings [35]. It has been speculated that positive expectations about the disease course, in the context of cancer, may give siblings a sense of cognitive control or mastery [35]. However, within the palliative care context, there can be no realistic expectation or hope for a cure. Nevertheless, an optimistic outlook may still be beneficial [32,36], and need not be tied to positive expectations of the course of the condition or disease. For some, optimism within the palliative care context may take the form of spirituality [37]. For others, optimism and hope in the context of palliative care may be goal-focused and associated with meaningful interactions with family and friends [37]. The specific nature of parental hopes in the context of having a child with advanced cancer has been documented [38], but less is known about optimism and hope among siblings. In other contexts, such as among carers working in the palliative care setting, optimism has been found to be associated with lower levels of perceived stress [36]. Carer optimism may be a powerful predictor of which caregivers are likely to respond more favorably to the caregiver role [39]. The role of optimism among healthy siblings of children with LLCs has not been well studied.

The trait positive affect refers to a general disposition towards positive emotions. Although the trait positive affect is a much broader construct than trait optimism, the two are likely to be related. Positive emotions may contribute to the implementation of cognitive strategies to reframe stressful situations in a more positive vein [40] and may lead to a more positive outlook. More generally, the importance of the positive affect has been explained with the broaden-and-build theory [41]. This theory holds that the positive affect broadens the range of behavioral outcomes during high distress situations through the increase of awareness, attention, and thoughts. Over time, this broadening process could allow personal resources such as social support and coping mechanisms to be built [15,41,42]. A study of healthy children with a brother or sister with a chronic physical disorder found the sibling negative affect to be associated with poorer adjustment outcomes [43]. In contrast, the development of warmth, compassion, and a positive outlook has been associated with more positive adjustment outcomes [15,44].

Psychological flexibility may be defined as the ability to respond appropriately to changing emotional circumstances [45]. Living with a brother or sister who has an LLC may be associated with frequent changes in emotional circumstances, associated with fluctuations in the child’s condition and disease progression. The ability to respond flexibly to these changes is likely to be associated with more favorable sibling outcomes. Although the construct of psychological flexibility is relatively new, having gained recognition in recent years within the context of acceptance and commitment therapy [46,47], earlier researchers have examined related constructs of adaptability [48] and emotional self-regulation [49] in families with a seriously ill child. In each of these studies, greater flexibility, adaptability, and ability to self-regulate emotions has been associated with more favorable outcomes among healthy family members.

Key sub-processes that have been associated with current conceptualizations of psychological flexibility are acceptance and mindfulness [42]. Acceptance involves individuals allowing themselves to experience unpleasant thoughts and emotions, rather than struggling to push them outside of consciousness. The related concept of mindfulness pertains to a mental state of awareness that involves bringing one’s complete attention to the internal and external experiences occurring in the present [50]. Both of these concepts have theoretical appeal and application in the context of siblings living with a brother or sister who has a serious medical condition. A qualitative study with siblings and parents of children with Duchenne muscular dystrophy found that some siblings reported finding acceptance of the situation to be helpful [15]. However, empirical investigation of these concepts has thus far been limited, particularly with siblings of children with LLCs or serious medical conditions.

### 5.2. Familial Resilience Factors

Given that resilience requires individuals to navigate their way towards health-sustaining resources, within the pediatric context, it is thus largely the role of the parents to make such resources available to their children. However, siblings living with a brother or sister with a serious medical condition or an LLC, parents, and the whole family are often living with considerable levels of stress, and the ability of parents or the family unit to support the healthy siblings may be compromised. Nevertheless, familial resources that are likely to be important for promoting child resilience include parental adjustment and family cohesion, as well as effective communication. Socio-economic status may also affect the family’s adaptation to adverse events, and may impact on the promotion of child and family resilience. Each of these factors will be considered in more detail. Literature regarding the role of birth order, sibling age, and the nature of the relationship between the sick and well siblings on the functioning and resilience of well siblings will also be reviewed.

Parents who are themselves better adjusted to the adverse situation are not only more likely to model effective coping skills, but are also more likely to have sufficient coping resources to support the well siblings, relative to parents who are poorly adjusted. Parental distress, depressed moods, and poorer well-being have been found to be significantly associated with poorer outcomes among the siblings of children with a range of chronic physical illnesses or conditions, or developmental disorders [17,43,51]. This may be due to poor parental modeling of coping behaviors, parental preoccupation with the sick child, low levels of parental acceptance and availability, or difficulties in adjusting family routines as required [7,15,24]. In contrast, there is some evidence to suggest that positive parental adaptation (e.g., low distress, acceptance of adversities and sibling’s emotions, appropriate affective expression) may lead to greater family satisfaction as perceived by siblings, thus having a positive impact on sibling adjustment [17,24,44].

Family cohesion is the emotional bonding that family members have towards one another [52]. A positive parent–child relationship and supportive family environment can help siblings adapt successfully to adverse events [51,53]. In particular, expressiveness, affection, shared decision-making, and problem-solving have been found to be important in contributing to good family functioning in times of adversities [44,51]. Conversely, lack of family cohesion has been found to be associated with sibling adjustment problems [17,24,51,54].

Family communication is well recognized as impacting on the well-being of children [55]. This is particularly likely to be the case when families are facing stressful situations with which they are unfamiliar, such as the serious illness of a parent [56] or the serious illness of a sibling [53]. In the context of living with a brother or sister who has a life-limiting condition, effective communication has been found to be positively correlated with sibling adjustment [27,54]. Open family communication helps siblings to better understand the situation, therefore minimizing misunderstandings and disappointment [14,15,19]. It has the potential to not only enable siblings to feel better informed about their brother or sister’s condition, but to enable the healthy siblings to share their concerns and seek and obtain emotional support when desired. One study found that children who reported receiving more communication about their brother or sister’s cancer also reported lower perceived impact of their brother or sister’s illness on their own life [57]. However, there are different levels of desire for information regarding the illness depending on age and the siblings’ own desire to know more [22,28,53].

Although well siblings often desire the attention of their parents [15], there are contradictory findings on the relationship between parental availability and sibling adjustment. Some papers have reported that reduced parental attention is associated with increased adjustment problems [6,15], whereas other studies have found little direct association between parental inattention and poorer siblings’ functioning [26]. It may be that some parents are able to find others to help attend to the various needs of healthy siblings, for example, grandparents, friends, and teachers [25], whereas others might not have similar support systems.

In a few studies, socioeconomic status of the family has been found to be positively associated with the psychological adjustment of siblings of seriously ill children [23]. Low family income in the context of childhood cancer has been found to be associated with increased siblings’ distress [24], whereas high family income has been linked to higher parent-reported siblings’ quality of life [23]. Socioeconomic status can be seen as a protective factor as it correlates with other family adaptation variables. For example, a stable financial income may overcome some of the practical challenges such as access to appropriate healthcare services and supports for the family [53].

Birth order and age difference between the well and unwell siblings may impact on how well siblings respond to the situation of having a brother or sister with serious medical problems [16,18,26,54,58]. Siblings closer in age to the unwell child have been found to have greater internalizing behaviors [54], possibly related to their closer involvement during disease progress. Another study found older siblings were at higher risk of adjustment problems [35], perhaps because of greater involvement in the care of the unwell child, or because of more significant losses of parental time and attention, as parents may have assumed that they were old enough to manage things themselves. Well siblings, especially older siblings, may also be called on to take on additional responsibilities within the family. Irrespective of the age of the sibling, there is considerable variability among well siblings with regard to their preferred level of involvement in caring for the unwell child [28,54]. Some siblings perceive involvement in caring for their sick brother or sister as important, helping them feeling included [28]. However, other siblings have expressed a preference to avoid caring responsibilities [54].

Findings are mixed with regard to the effect that the closeness of the relationship between the well and unwell sibling may have on the adjustment of the well sibling, however, studies have been with different patient populations. One study found that siblings of cancer patients who reported a closer relationship with the unwell child tended to have poorer adjustment, possibly because of a heightened appreciation of distress or suffering experienced by the sick child [16]. In contrast, other studies have found a closer sibling relationship to be associated with more positive adjustment outcomes for children with a brother or sister with Down syndrome [17,44], possibly because of greater empathy and acceptance. These discrepancies may be because ofthe fact that siblings of cancer patients may have been required to make quite a sudden and significant adjustment following the onset of the disease, whereas siblings with a brother or sister with Down syndrome have typically had more time to adjust.

In the context of having a sibling with a congenital disease, older well siblings with a larger age gap may have had a relatively ‘normal’ environment throughout their early development, before the birth of the sick child [26,58]. While on the one hand, this may result in the need to make significant changes to routines to which they are accustomed, it also means that they may have benefited from greater stability and lower family stress levels during the early years of their life.

### 5.3. External/Social Support Resilience Factors

The availability of appropriate external supports, outside of the core family unit, may help to minimize the negative impact of parental stress and unavailability on well siblings. Grandparents and extended family, teachers, counsellors, friends, and various home support services are able to provide the family and the well siblings with much needed help that parents may be unable to offer at certain times. Interviews with siblings of children with juvenile idiopathic arthritis found that external support, like having sleep-overs at grandparents’ homes, playing with cousins, and getting help with shopping, helped buffer some of the negative emotions that siblings experienced at times [18]. Social support from the wider community, such as peers, teachers, mentors, or religious groups, has been reported to enable siblings to vent feelings, seek emotional and academic help when parents are occupied, reduce loneliness, enhance self-efficacy, and enhance self-esteem [25,44,53,57,59]. The ability to seek help from other platforms is extremely important, especially when siblings do not want to overburden parents but yet are in need of support.

The ability of families who have a child with an LLC to access and utilize respite care has been found to be important for the well-being of the whole family [23]. Pediatric respite care is the provision of care to children with LLCs and their families by qualified caregivers and may occur within or outside of the home. It allows families to take ‘time off’ from the demands of patient care and to rest and rejuvenate intermittently, this being especially important for parents to help find time to care for themselves and their healthy children [60]. Notably, self-reported quality of life has been found to be higher among siblings of children with LLCs if their families accessed respite services relative to those who did not access respite care [23]. It is relevant to note that respite service type and availability vary considerably, along with the models of how such services are managed and funded. There are many factors that impact a family’s ability to access respite care that meets the individual needs of the child and family; further consideration of these is beyond the scope of this paper.

## 6. Future Directions for Research and Clinical Practice

Given the limited number of studies focusing on the functioning of children with a brother or sister with an LLC, the current paper has drawn considerably from the contexts of other serious illnesses (e.g., cancer) and chronic conditions (e.g., juvenile idiopathic arthritis, diabetes, autism spectrum disorders). More research is needed in the context of having a sibling with an LLC. Taking a longitudinal approach may help identify factors contributing to sibling functioning over time. In light of the relatively low numbers of children with LLCs, multi-site collaborations would enhance the ability to further clarify individual, familial, and broader social factors associated with sibling resilience. Furthermore, an objective evaluation of the efficacy of existing services for siblings and families is needed to ensure that resource allocation is guided by best available evidence, and to help shape the development of new services. As open familial communication patterns have generally been associated with more favourable sibling functioning [14,15,19], more research is needed into how best to promote open and effective familial communication. However, the question remains as to whether open communication leads to better sibling outcomes, or whether better functioning families are more likely to communicate openly. Future research should also consider how best to achieve an optimal balance between the extent to which a sibling desires information about their brother or sister’s condition, and a parent’s readiness and ability to provide this information.

Because of the nature of LLCs, pediatric palliative care teams may have long-term relationships with patients and their families. In clinical practice, this may afford them with an opportunity to carry out research in this field to identify individual, familial, and external/social resilience factors that may be utilized to the family’s advantage and to the benefit of the well siblings. Moreover, an understanding of possible modifiable resilience factors may enable clinicians in paediatric palliative care to help foster specific resources that may help families, including the well siblings, in how they respond to future challenges, such as the deteriorating condition of the unwell child.

It is important to acknowledge that studies in the field of pediatric palliative care are highly susceptible to self-selection and non-invitation biases. Given that research participation is voluntary, siblings (or families) who are highly distressed may be less likely to participate [61] unless they perceive that research participation offers them some sort of additional support. Moreover, many studies within this literature are compromised because of the non-invitation “gate-keepers” bias [62], whereby health professionals may avoid recruiting certain families for non-specified reasons, such as perceiving that a family is already over-burdened or highly distressed. These biases may limit the generalizability of published findings.

## 7. Conclusions

Although it is generally recognized that the holistic care of children with LLCs involves the consideration of siblings as part of the family unit, research into the experiences, outcomes, and needs of these siblings is limited. There is considerable variation in the adjustment and functioning of these healthy siblings, with some showing significantly more adaptive and resilient responses. A range of individual difference factors, familial factors, and external social factors have been found to be associated with greater sibling resilience in the context of living with a seriously ill brother or sister. Further exploration of these factors is needed within the context of LLCs, and consideration of whether sibling resilience may be enhanced by promoting certain factors or conditions is warranted.
